# Association between Lifestyle Factors and Quality-Adjusted Life Years in the EPIC-NL Cohort

**DOI:** 10.1371/journal.pone.0111480

**Published:** 2014-11-04

**Authors:** Heidi P. Fransen, Anne M. May, Joline W. J. Beulens, Ellen A. Struijk, G. Ardine de Wit, Jolanda M. A. Boer, N. Charlotte Onland-Moret, Jeljer Hoekstra, Yvonne T. van der Schouw, H. Bas Bueno-de-Mesquita, Petra H. M. Peeters

**Affiliations:** 1 Julius Center for Health Sciences and Primary Care, University Medical Center Utrecht, Utrecht, the Netherlands; 2 National Institute for Public Health and the Environment (RIVM), Bilthoven, The Netherlands; 3 Department of Gastroenterology and Hepatology, University Medical Center Utrecht, Utrecht, the Netherlands; 4 School of Public Health, Imperial College London, London, United Kingdom; 5 Dt. of Social & Preventive Medicine, Faculty of Medicine, University of Malaya, Kuala Lumpur, Malaysia; Sookmyung Women's University, Republic of Korea

## Abstract

The aim of our study was to relate four modifiable lifestyle factors (smoking status, body mass index, physical activity and diet) to health expectancy, using quality-adjusted life years (QALYs) in a prospective cohort study. Data of the prospective EPIC-NL study were used, including 33,066 healthy men and women aged 20–70 years at baseline (1993–7), followed until 31-12-2007 for occurrence of disease and death. Smoking status, body mass index, physical activity and adherence to a Mediterranean-style diet (excluding alcohol) were investigated separately and combined into a healthy lifestyle score, ranging from 0 to 4. QALYs were used as summary measure of healthy life expectancy, combining a person's life expectancy with a weight for quality of life when having a chronic disease. For lifestyle factors analyzed separately the number of years living longer in good health varied from 0.12 year to 0.84 year, after adjusting for covariates. A combination of the four lifestyle factors was positively associated with higher QALYs (P-trend <0.0001). A healthy lifestyle score of 4 compared to a score of 0 was associated with almost a 2 years longer life in good health (1.75 QALYs [95% CI 1.37, 2.14]).

## Introduction

Chronic diseases are important causes of death and disability worldwide. Almost 54% of the global burden of disease is due to non-communicable diseases such as cardiovascular diseases and cancer [1]. Nowadays in high income countries, total disease burden is more affected by the years lived with disability than by premature deaths, as the number of premature deaths due to several chronic diseases decreased in the past decades [1], [2]. Several known risk factors for these chronic diseases are modifiable, including diet, smoking behavior, weight and physical activity [3]. These factors are leading contributors to the global disease burden [2].

The association of modifiable lifestyle factors with either mortality and morbidity has been studied before [4]–[7]. We set out to study the association between a combination of modifiable lifestyle factors and total disease burden using QALYs, a summary health measure that combines life expectancy (mortality) with quality of life (morbidity). QALYs incorporate the effect of different diseases into one outcome measure of health. It provides a more complete picture of the effect of risk factors on population health than using only morbidity and mortality. The QALY was originally developed for use in economic evaluations of health interventions and is generally used as a measure of health gain when comparing interventions [8]. One QALY equals one year in optimal health, while zero QALY equals death. Utility weights are applied to represent the reduction in quality of life attributable to having a certain chronic disease.

Two previous studies used QALYs to study the association between lifestyle factors and burden of disease [9], [10]. One [9] investigated the effect of smoking, physical activity or obesity separately on quality-adjusted life expectancy in the Danish general population. They combined information from life tables with observed age-specific prevalence rates of specific health states to calculate healthy life expectancies (Sullivan's method) [11]. Smoking showed the largest impact, followed by physical inactivity and obesity. However, clustering of unhealthy behavior within persons was not taken into account in this study. The other [10] combined lifestyle behaviors and related this to QALYs in a sample of the general population, the EPIC-Norfolk cohort. People with higher health behavior scores had significantly higher QALYs. The study was cross-sectional in that it assessed lifestyle behaviors at baseline in relation to baseline QALY weights. Neither of these studies prospectively followed participants for disease occurrence. The aim of the present study is to relate four modifiable lifestyle factors, smoking status, BMI, physical activity, and adherence to a Mediterranean-style diet, to overall healthy life expectancy, using QALYs. In addition, clustering of unhealthy lifestyle was evaluated by combining lifestyle factors into a healthy lifestyle score. For this purpose, QALY weights were applied to a large cohort that prospectively followed participants for occurrence of chronic diseases.

## Methods

### Study population

The association between lifestyle factors and QALYs was investigated in the EPIC-NL study [12]. This prospective cohort study combines the two Dutch cohorts of the European Prospective Investigation into Cancer and Nutrition, i.e. EPIC-MORGEN and EPIC-Prospect [13]. 40,011 men and women aged 20–70 years were recruited between 1993 and 1997. At baseline all participants filled out a general questionnaire and a validated food frequency questionnaire, and underwent a physical examination. All participants provided written informed consent before study inclusion. The study complies with the Declaration of Helsinki and was approved by the Institutional Review Board of the University Medical Center Utrecht and the Medical Ethical Committee of TNO Nutrition and Food Research.

Participants were followed for the occurrence of incident diseases by linkage to the Dutch Cancer Registry and the hospital discharge diagnosis database of the National Medical Registry. The National Medical Registry includes information on hospitalized patients; visits to out-patient clinics are not included. Chronic diseases that were identified during follow-up included: diabetes mellitus, stroke, myocardial infarction, other heart conditions, asthma, Chronic Obstructive Pulmonary Disease, osteoarthritis, rheumatoid arthritis, Parkinson's disease and cancer (for this study subdivided into uterus-, bladder-, breast-, colorectal-, skin-, lung-, lymphoma, stomach-, kidney-, prostate-, and other cancer). Diabetes Mellitus was verified using information of the general practitioner or pharmacist [14]. Information on date and cause of death was obtained from linkage with municipal registries and Statistics Netherlands. Follow-up information was available until December 31, 2007. Mean follow-up was 12.4 years.

Exclusion criteria were: no written consent for linkage with the registries (n = 2879), having one of the investigated chronic diseases at recruitment (this may influence lifestyle at baseline, n = 3625), missing dietary information at baseline (n = 142), and extreme energy intake levels (being in the top or bottom 0.5% of the ratio of reported energy intake over estimated energy requirement, n = 299). Finally, 33,066 participants were included in the analysis.

### QALY calculation

The QALY combines information on both life expectancy and quality of life. Years lived with a disease are weighed based on ‘utility weights’ that vary between 0 and 1. One full year in optimal health equals 1 QALY and death equals 0 QALYs. The utility weights that are used to weigh years lived in a suboptimal health state are in general derived from individual patient health status data, combined with preferences from the general population concerning those specific health states [15]. Standardized instruments, such as the EQ-5D instrument [16], have been developed to attach utility values to different health states. EQ-5D health states have been valued by a representative sample of the Dutch population, which resulted in a validated algorithm [15].

To be able to calculate utility weights for specific diseases, information on quality of life is needed from diseased subjects. This information was not available in EPIC-NL. We developed a prediction model for disease-specific utility weights, using data of the second Dutch National Survey of General Practice (DNSGP-2) [17]. Details of this prediction model are given in appendix S1, Table S1 in appendix S1 and Table S2 in appendix S1. Based on this model utility weights for different diseases, age, sex, education and working status were obtained.

QALYs were estimated for three periods in a person's life: 1. from birth to study recruitment, 2. during the study, and 3. from the end of study (December 2007) until the expected end of life. QALY weights before study entry were based on the participant's gender and age only. Years during the study period were weighted based on disease status and on gender, age, having a paid job and educational level (appendix S1). For each individual all QALYs were summed over the study period. Observed follow-up ended on December 31, 2007 or earlier for death and loss to follow-up. For the calculation of QALYs for the period after study end, we assumed that participants kept the disease state as observed at the end of follow-up until expected date of death. Life expectancy was determined using the participants' age at end of follow-up and the reference year 2007 for national life expectancy statistics. Statistics Netherlands provided age- and gender-specific life expectancy tables [18]. For those lost to follow-up before 2007, calendar year of loss to follow up was used as the reference year for life expectancy calculation. For participants who died before 2007 the QALY weight was set to zero from that date onwards.

### Lifestyle factors

Smoking status was defined at recruitment as current, former or never smoker. BMI was calculated from measured height and weight and categorized into normal weight (<25 kg/m^2^), overweight (25–30 kg/m^2^) or obese (≥30 kg/m^2^). Physical activity level was assessed in the general questionnaire and categorized according to the validated Cambridge Physical Activity Index (CPAI) in inactive, moderately inactive, moderately active or active [19]. Usual food intake as measured by the food frequency questionnaire [20] was used to determine a modified Mediterranean Diet Score (mMDS) [21], with the exception that alcohol consumption was not included in the score [22]. However, adjustments for alcohol consumption were made in the analyses. Sex-specific median consumption of eight dietary components was scored for the mMDS, i.e. consumption of fruit, vegetables, legumes, fish, cereals, meat and dairy products and the unsaturated to saturated fat ratio, and summed into a total score that ranged from 0 (minimal adherence) to 8 (maximal adherence). The sum score was categorized into low (0–2), moderate (3–5) and high (6–8) adherence to the mMDS for analysis.

### Healthy lifestyle score

In addition to separate analysis of smoking status, BMI, physical activity level and adherence to a Mediterranean-style diet, they were combined into one pragmatic healthy lifestyle score to investigate their combined effect. For the healthy lifestyle score the last three categories of physical activity level, moderately inactive, moderately active and active, were combined into ‘being active’. The mMDS was dichotomized into low (0–4) or high (5–8) adherence. Participants scored one point for each of the following lifestyle categories: never smoking, having a normal weight, being physically active and having high adherence to the Mediterranean diet. Hence, the healthy lifestyle score ranged from 0 (unhealthy lifestyle) to 4 (healthy lifestyle). Moderate alcohol consumption was not included as a healthy lifestyle in the score. In most observational studies moderate alcohol intake is associated with decreased risks of cardiovascular disease and diabetes, but any consumption of alcohol is a risk factor for some cancers [23]. Furthermore, any alcohol use may increase the risk of binge drinking and alcohol abuse causes health-related harms and higher disease burden [2], [24]. Therefore, as we have done previously [22], we decided not to include alcohol consumption in the score, but adjusted for it in the analyses.

### Covariates

Age, gender, educational level, alcohol consumption and energy intake were assessed at recruitment. Educational level was categorized into low (lower vocational training or primary school), middle (secondary school or intermediate vocational training) and higher education (higher vocational training or university). Alcohol intake was included in the model in 7 categories: 0 g/day, ≤6 g/day, 6-≤12 g/day, 12-≤24 g/day, 24-≤60 g/day,>60 g/day (women) or 60-≤96 g/day (men) and more than 96 g/day (men). Energy intake (kcal/day) was used as a continuous variable.

### Statistical analysis

Missing data on smoking status, BMI, CPAI, educational level and working status were imputed using single imputation regression modeling (SPSS-MVA). Percentage of missing's ranged from 0.1 to 3% for the different variables. Information on physical activity was missing in 14% of the participants, as in the first year of the EPIC-MORGEN study (1993) physical activity was not assessed with the EPIC questionnaire. Population characteristics according to healthy lifestyle score were presented as mean and standard deviation, median or as a percentage. The association of the separate lifestyle factors or the healthy lifestyle score with QALYs was estimated by linear regression. Regression coefficients and 95% confidence intervals are presented. Additionally, adjustments were made for age at recruitment, gender, educational level, alcohol and energy intake. Analyses for the separate lifestyle factors were additionally adjusted for the other lifestyle factors in the score. All analyses were stratified for cohort. A linear P for trend was computed by including the lifestyle factor or healthy lifestyle score as a continuous variable. Effect modification by gender, age, and educational level was explored by including interaction terms in the model. We performed sensitivity analyses to investigate the effect of excluding participants with a BMI <18 and the effect of including waist circumference instead of BMI in the healthy lifestyle score (1 point if waist is below 94 cm for males or below 80 cm for females). Some diseases that were included in the utility weight model had large confidence intervals around the regression coefficients (see Table S2 in Appendix S1). We repeated the analysis and used the lower and upper bounds of the 95% confidence intervals instead of the mean values. Statistical analyses were conducted using SAS 9.2 (SAS Institute, Cary, US).

## Results

Table 1 shows characteristics of the study population according to healthy lifestyle score. Participants with the highest score were more often female, young, working and higher educated than participants with a low score.

**Table 1:** Characteristics of 33,066 healthy EPIC-NL participants according to a healthy lifestyle score.

	Healthy lifestyle score				
	0	1	2	3	4
	n = 621 (2%)	n = 7192 (22%)	n = 13824 (42%)	n = 9215 (28%)	n = 2214 (7%)
Sex (male %)	37	31	25	23	23
Age at study entry (mean(sd))	53.0(9.4)	50.8(10.3)	49.3(11.7)	47.1(12.7)	45.2(13.3)
Working (yes %)	36	57	63	68	72
Higher educational level (%)	11	14	18	27	35
Alcohol intake (median(IQR), g/day)	5 (0–23)	6 (1–20)	5 (1–16)	5 (1–14)	4 (1–11)
Total energy intake (mean(sd), kcal/day)	1976(584)	2044(609)	2057(614)	2082(610)	2113(590)
Never smokers (%)	0^a^	5	32	62	100^a^
BMI <25 kg/m^2^ (%)	0^a^	6	43	75	100^a^
Physically active (%)	0^a^	85	96	99	100^a^
mMDS score 5–8 (%)	0^a^	4	28	63	100^a^

a By definition (a score of 4 implies all group members are never smoker, physically active and have a normal BMI and a high mMDS score).

Table 2 shows results for the individual lifestyle factors. The regression coefficients can be interpreted as the higher (or lower) number of QALYs that an individual has compared to individuals with score zero. Compared to current smokers, never smokers had 0.84 healthy years longer (95% CI: 0.72,0.95), whereas past smokers showed 0.65 (95% CI: 0.53,0.77) QALYs. Normal weight was associated with 0.44 (95% CI: 0.29,0.59) higher QALYs compared to obesity. Participants who were physically active had 0.73 (95% CI: 0.54,0.92) higher QALYs compared to inactive participants. Adherence to the mMDS was significantly associated with higher QALYs when added as a continuous variable to the model (p = 0.01).

**Table 2:** Regression Coefficients for the relation between separate lifestyle factors and QALYs (N = 33,066).

	N	Mean QALY	Crude	P for trend	Adjusted^a^	P for trend
**Smoking status**						
Current	10035	74.16	reference	<0.0001	reference	<0.0001
Past	10251	75.15	0.99 (0.87, 1.11)		0.65 (0.53, 0.77)	
Never	12780	75.32	1.16 (1.05, 1.28)		0.84 (0.72, 0.95)	
**BMI category**						
Obese *≥30 kg/m^2^*	4372	74.64	reference	<0.0001	reference	<0.0001
Overweight *25*–*30 kg/m^2^*	13142	74.83	0.19 (0.04, 0.34)		0.25 (0.10, 0.39)	
Normal weight*<25 kg/m^2^*	15552	75.06	0.43 (0.28, 0.57)		0.44 (0.29, 0.59)	
**Physical activity level**						
Inactive	2273	74.12	reference	<0.0001	reference	<0.0001
Moderately inactive	8038	74.89	0.76 (0.56, 0.97)		0.48 (0.29, 0.68)	
Moderately active	8696	74.98	0.86 (0.66, 1.06)		0.57 (0.37, 0.77)	
Active	14059	75.02	0.90 (0.70, 1.09)		0.73 (0.54, 0.92)	
**mMDS**						
0–2	5159	74.63	reference	<0.0001	reference	0.01
3–5	22673	74.93	0.30 (0.17, 0.43)		0.11 (−0.02, 0.24)	
6–8	5234	75.12	0.48 (0.32, 0.65)		0.12 (−0.05, 0.28)	

a Adjusted for age at baseline, gender, educational level, alcohol and energy intake and the other lifestyle factors.

Participants who reported to comply with all four lifestyle factors had statistically significantly more QALYs compared to participants that did not comply (1.75; 95% CI: 1.37,2.13), i.e. they lived almost 2 years longer in good health (table 3). The greatest statistically significant association between QALY and lifestyle was observed for a change in healthy lifestyle score from 0 to 1 (0.97; 95% CI: 0.62,1.32), while the association was lower for changes from 3 to 4 (0.17; 95% CI: −0.03,0.37).

**Table 3:** Regression Coefficients for the relation between healthy lifestyle score and QALYs (N = 33,066).

	N	mean QALY	Adjusted^a^	
Healthy lifestyle score				P for linear trend
0	621	73.40	ref	<0.0001
1	7192	74.41	0.97 (0.62, 1.32)	
2	13824	74.93	1.35 (1.01, 1.70)	
3	9215	75.25	1.58 (1.23, 1.93)	
4	2214	75.48	1.75 (1.37, 2.13)	

a Adjusted for age at baseline, gender, educational level, alcohol and energy intake.

Sensitivity analysis, excluding participants with a BMI below 18 (n = 187) or including waist circumference instead of BMI in the score, did not alter these results. Also, using the lower and upper bound 95% CI for the EQ-5D utility weight analysis did not affect the results (data not shown). Interactions between healthy lifestyle score and gender, age or educational level were not significant.

## Discussion

In this large prospective study, nonsmoking, normal BMI, higher physical activity level and adherence to a Mediterranean-style diet were all associated with a longer healthy life. Being non-smoker and physically active were associated with the highest number of quality-adjusted life years. Furthermore, a combination of the four lifestyle factors was associated with a significantly longer life in good health. People who reported to have never smoked, have a normal weight, are physically active and who adhere to the mMDS lived on average almost two healthy years of life longer compared to people with a less healthy lifestyle.

Strengths of our study are its prospective design, large sample size and the use of a summary health measure to investigate healthy life expectancy. The use of QALYs allowed us to investigate the effect of lifestyle factors on overall health. At the end of the follow-up period (2007), 20% of the cohort suffered from at least one disease and only 4.5% died. Ideally, the cohort should be followed until it is extinct. In the present study, the true beneficial effects of lifestyle factors may therefore have been underestimated. Participants were relatively healthy at study entry, because participants with a prevalent disease at baseline were excluded to rule out the risk of reverse causation. Furthermore, presumably, people with a disease live shorter than the average life expectancy and people without disease live longer than the average life expectancy. Different life expectancies according to disease status were not taken into account, as these data were not available. Moreover, after follow-up was ended, it was not possible to account for new diseases, which probably will develop more frequently in people with an unhealthy lifestyle. That would result in larger differences in QALYs between participants having a healthy or an unhealthy lifestyle. Therefore, our QALYs observed with a healthy lifestyle are likely to be a minimum estimate of the true association.

We could not use our study population to derive the utility weights. Therefore, information on utility weights of the DNSGP-2 study was used [17]. As the DNSGP-2 consists of a representative sample of the Dutch GP population and as health-related quality of life was measured through the standardized EQ-5D instrument, we believe that using utility weights obtained from this population is justified. For some diseases, the 95% CI of EQ-5D utility weights were quite large. We therefore investigated the effect of using the lower and upper bound of the 95% CI instead of the mean utility weight in our analysis. This did not alter our results. Regarding endpoint assessment, not all possible lifestyle related diseases could be included. However, we included the most important chronic diseases. Furthermore, several disease data were based on hospital discharge data, while some of the diseases, such as COPD and rheumatoid arthritis, not often require hospitalization. This could have resulted in an underestimation of incidence rates for these diseases. Presuming that these diseases develop more in people with an unhealthy lifestyle, it would result in an underestimation of the QALY difference. Another limitation is that lifestyle factors were only assessed at baseline and possible behavioral changes during follow-up were not taken into account.

Other studies investigated lifestyle factors and health expectancy cross-sectional or applied Sullivan's life table approach using population-based QALYs [9], [10]. In our study we computed individual QALYs and adjusted for confounding. Moreover, our study participants were prospectively followed for disease occurrence and their utility weight was adjusted accordingly, i.e. every time a disease occurred the quality of life was adjusted. For most diseases utility weights are lower in the first year of diagnosis. Assuming that disease utilities stay constant from the start of the disease onwards seems incorrect and may lead to an overestimation of QALYs associated with a healthy lifestyle. Our results are in line with the results of Brønnum-Hansen *et al.* [9] who also reported the greatest benefit of never smoking and being physically active on QALYs. For heavy smoking men almost 10 fewer healthy life years (QALYs) were expected than for never smokers, while we observed only 0.84 fewer QALYs in smokers compared to non-smokers. However, the methods they used differ from our methods: we cannot directly compare the results. They used Sullivan's life table approach together with average age-specific QALY weights to calculate expected QALYs per risk factor exposure level. In Sullivan's life table approach expected QALYs are calculated per risk-factor exposure level and each exposure level results in a different life expectancy. The larger QALY gains found by Brønnum-Hansen *et al.* might be explained by the fact that in the present study the true effect is still underestimated, as discussed above.

Myint *et al*. investigated QALYs and lifestyle behavior [10]. They showed that having four healthy behaviors (non-smoking, not physically inactive, drinking 1 to 14 units of alcohol/week and consuming 5 or more servings of fruit and vegetables/week) was related with 1 more healthy year compared to participants with 0 healthy behaviors. In our study almost 2 healthy years were related with adhering to four healthy lifestyle factors. This difference may be explained by the difference in components of the score and the different calculation in QALYs. Myinth *et al.* calculated QALYs until the end of follow-up, while in the present study QALYs were calculated until the end of life by assuming a status quo from end of follow-up to end of life. Finally, Klijs *et al.* studied effects of BMI, smoking and alcohol consumption on years lived with disability and mortality, without using the concept of QALYs. They used the Sullivan life table method [25] and showed that smoking affected mortality more than morbidity, and the other way around for obesity. This supports our method combining mortality and morbidity to investigate total disease burden.

In conclusion, in this prospective study never smoking, having a normal weight, being physically active and adherent to a Mediterranean-style diet were positively associated with healthy life expectancy. The combination of these four lifestyle factors was associated with almost 2 years of life longer in good health. Our findings implicate that public programs aiming at improving health could benefit from targeting at a cluster of modifiable lifestyle factors.

## Supporting Information

Appendix S1 Deriving utility weights.
(DOC)
