# Supplementary material for: Association between Lifestyle Factors and Quality-Adjusted Life Years in the EPIC-NL Cohort
Source: PLoS One. 2014 Nov 4;9(11):e111480. doi: 10.1371/journal.pone.0111480 (PMC4219750; doi:10.1371/journal.pone.0111480)
Supplement: Appendix S1 — Deriving utility weights. (DOC) [file pone.0111480.s001.doc]

## Appendix S1: Deriving utility weights

Baseline information on quality of life (SF-36 questionnaire) was available for a sample of the EPIC-NL study, but the number of prevalent diseases at baseline within EPIC-NL was too low to estimate utility values associated with chronic disease. Therefore, we developed a prediction model for utility weights for different diseases, using data of the second Dutch National Survey of General Practice (DNSGP-2).

### Study population

The DNSGP-2 provides information on disease incidence in 2001 as well as educational level, working status, age and gender of patients registered at 104 general practitioners in the
Netherlands [1]. In a 5% random sample of this population (n=12,699) a health interview was taken, including questions on chronic disease and health-related quality of life (EQ-5D questionnaire). Details of the study have been reported previously [1]. Table S1 shows some characteristics of the study population.

Information on self-reported chronic disease prevalence as well as health-related quality of life was available for 9654 patients. For types of cancer that occurred in more than 10 patients in this population (uterus, bladder, breast, colorectal, skin, lung, lymphoma, stomach, kidney and prostate cancer) separate utility indices were derived. Cancers occurring less than 10 times in this population were grouped into ‘cancer, other type’. Parkinson’s disease was not included in the health interview. We used information from the general practitioner’s registration to identify prevalent Parkinson’s patients.

Health-related quality of life was measured through the standardized EQ-5D instrument of the Euroqol group [2]. The EQ-5D has been validated for use in the Netherlands. A validated algorithm [3] attaching utility weights to each of the 243 health states that can be described with EQ-5D was used. Individual factors, such as educational level, age and gender, are taken into account when estimating someone’s health related quality of life. Therefore, we derived a model to predict utility weights, using patient characteristics and several chronic diseases as predictors.

Patients with missing data on all five EQ-5D questions or on all self-reported chronic diseases were excluded from the analysis (n=25). Missing values in some of the EQ-5D questions and in the questions on chronic diseases, work and educational level were imputed using multiple imputation (SPSS).

Model for utility weights A linear model was used to derive prediction equations with utility scores as the dependent variable. Predictors in the model were gender, age, working status (categorized into working or not working), educational level (categorized into low, middle and higher education, based on the classification used in the EPIC-NL study [4] ), and the chronic diseases diabetes, stroke, myocardial infarction (MI), other heart conditions, asthma/Chronic Obstructive Pulmonary Disease, osteoarthritis, rheumatoid arthritis, Parkinson’s disease and cancer (uterus, bladder, breast, colorectal, skin, lung, lymphoma, stomach, kidney, prostate and other cancer).
For some diseases (cancer, stroke and MI) quality of life during disease progression or after diagnosis changes. In the model based on time of diagnosis these diseases were split into ‘within 12 months’ or ‘more than a year ago’. For other heart conditions only information on the 12 months preceding the interview was available. We assumed that the utility weight for a patient with other heart conditions diagnosed more than a year ago was comparable to the weight of a patient who had a MI more than one year ago.

Statistical analysis
The utility weight prediction model was built in IBM SPSS Statistics for Windows, Version 20 (IBM Corp., Armonk, NY), using linear regression with the method ‘enter’. The regression coefficients of the model and 95% confidence intervals are presented in Table S2. These utility weights were applied to our study population each time a chronic disease occurred to prospectively calculate QALYs for each participant.
For example: the utility weight for one year of a 50 year old man, with a higher educational level, a job, diabetes mellitus and a myocardial infarction event less than 12 months ago (first year) is:
0.873 (constant) + 0.022 (male gender) + 50*0.000154 (age) + 0.039 (work) + 0.024 (educational level) -0.028 (diabetes mellitus) -0.004 (myocardial infarction, first year) = 0.9337.
If this person develops bladder cancer in the next year, his utility weight for that year will be:
0.873 (constant) + 0.022 (male gender) + 51*0.000154 (age) + 0.039 (work) + 0.024 (educational level) -0.028 (diabetes mellitus) -0.026 (myocardial infarction, > 1year) – 0.056 (cancer, first year) = 0.8559

References appendix S1

1. Westert GP, Schellevis FG, de Bakker DH, Groenewegen PP, Bensing JM, et al. (2005)
Monitoring health inequalities through general practice: the Second Dutch National Survey of General Practice. Eur J Public Health 15:59-65.

2. Brooks R. (1996) EuroQol: the current state of play. Health Policy 37:53-72.

3. Lamers LM, Stalmeier PF, McDonnell J, Krabbe PF, van Busschbach JJ. (2005) [Measuring the quality of life in economic evaluations: the Dutch EQ-5D tariff]. Nederlands tijdschrift voor geneeskunde 149:1574-8.

4. Beulens JW, Monninkhof EM, Verschuren WM, van der Schouw YT, Smit J, et al. (2010) Cohort profile: the EPIC-NL study. Int J Epidemiol 39:1170-8.

Table S1: Baseline characteristics of study population DNSGP-2 (N=9654).

|  | **DNSGP-2 (N=9654)** |
| --- | --- |
| **Gender (% males)** | 45% |
| **Age in years (mean(sd))** | 48.9(17.0) |
| **Working (%)** | 50% |
| **Higher educational level (%)** | 21% |

Table S2: Mean regression coefficients (95% confidence interval) for EQ-5D utility weights derived from 9,654 participants of the Dutch National Survey of General Practice.

|  | **EQ-5D utility weight** | |
| --- | --- | --- |
|  | mean | (95% CI) |
| Constant | 0.873 | (0.860, 0.887) |
| Gender (male) | 0.022 | (0.016, 0.029) |
| Age (per year) | 0.000154 | (-0.000074, 0.000382) |
| Paid Work | 0.039 | (0.031, 0.046) |
| Educational level (middle) | 0.020 | (0.013, 0.028) |
| Educational level (high) | 0.024 | (0.016, 0.032) |
| Diabetes Mellitus | -0.028 | (-0.043, -0.012) |
| Stroke, > 1 year ^a^ | -0.069 | (-0.091, -0.047) |
| Stroke, first year ^a^ | -0.153 | (-0.201, -0.105) |
| Myocardial infarction, > 1 year | -0.026 | (-0.044, -0.008) |
| Myocardial infarction, first year | -0.004 | (-0.057, 0.048) |
| Other heart condition, first year | -0.069 | (-0.090, -0.048) |
| Asthma/COPD | -0.050 | (-0.062, -0.038) |
| Osteoarthritis | -0.095 | (-0.105, -0.085) |
| Rheumatoid arthritis | -0.097 | (-0.112, -0.082) |
| Parkinson’s disease | -0.307 | (-0.399, -0.214) |
| **Cancers** |  |  |
| Uterus, > 1 year | -0.024 | (-0.063, 0.015) |
| Bladder, > 1 year | 0.052 | (-0.003, 0.107) |
| Breast, > 1 year | -0.020 | (-0.055, 0.016) |
| Colorectal, > 1 year | -0.055 | (-0.102, -0.009) |
| Skin, > 1 year | -0.019 | (-0.048, 0.010) |
| Lung, > 1 year | 0.012 | (-0.085, 0.109) |
| Lymfnode, > 1 year | 0.053 | (-0.006, 0.111) |
| Stomach, > 1 year | -0.047 | (-0.097, 0.002) |
| Kidney, > 1 year | 0.066 | (0.014, 0.117) |
| Prostate, > 1 year | -0.009 | (-0.079, 0.061) |
| Other cancer, > 1 year | -0.000245 | (-0.040, 0.040) |
| All Cancer, first year | -0.056 | (-0.083, -0.029) |

^a^ ‘first year’ = diagnosed within one year preceding the interview; ‘> 1 year’ excludes the patients who were diagnosed within one year preceding the interview
